# Treatment of Seasonal Affective Disorder. The efficacy of Light therapy

**DOI:** 10.1192/j.eurpsy.2023.1262

**Published:** 2023-07-19

**Authors:** T. Jupe, I. Giannopoulos, B. Zenelaj, E. Myslimi

**Affiliations:** 1 Psychiatric Hospital of Attica, Athens, Greece; 2 National Center for Children Treatment and Rehabilitation; 3 Freelancer psychiatrist, Tirane, Albania

## Abstract

**Introduction:**

Seasonal affective disorder (SAD) is a type of depression that comes and goes in a seasonal pattern. Symptoms of SAD can include: a persistent low mood, a loss of pleasure or interest in normal everyday activities, irritability, feelings of despair, guilt and worthlessness, feeling lethargic (lacking in energy) and sleepy during the day, sleeping for longer than normal and finding it hard to get up in the morning, craving carbohydrates and gaining weight, difficulty concentrating.

**Objectives:**

The purpose of this study was to evaluate the response to different therapeutic interventions of seasonal depression

**Methods:**

Α biographical review was performed using the PubMED platform. All relevant articles were found using the keywords: seasonal affective disorder, treatment, and winter depression.

**Results:**

The main treatments are: lifestyle measures – including getting as much natural sunlight as possible, exercising regularly and managing your stress levels, light therapy – where a special lamp called a light box is used to simulate exposure to sunlight, talking therapies – such as cognitive behavioral therapy (CBT) or counseling, antidepressant medicine – such as selective serotonin reuptake inhibitors (SSRIs)

**Conclusions:**

Light therapy can be a very effective treatment for SAD, with most seeing an improvement of symptoms within the first week. A powerful lamp that replicates natural light, high-quality light boxes are recommended as they allow patients to spend a shorter time (up to 30 minutes at a time) using them.

**Disclosure of Interest:**

None Declared

